# Development of Mobile Software “SRCardioCare” Prototype for Implementing Home-Based Exercise Program Among Patients After Adult Cardiac Surgical Revascularization: Qualitative Feasibility Study

**DOI:** 10.2196/69197

**Published:** 2026-02-23

**Authors:** Ajith Kumar Pichai, A Sathya, Thillaigovindarajan SenthilKumar, Sadhanandham Shanmugasundaram, R Karthik

**Affiliations:** 1 Department of Cardiopulmonary Physiotherapy, Sri Ramachandra Faculty of Physiotherapy, Sri Ramachandra Institute of Higher Education and Research (DU), No.1, Sri Ramachandra Nagar, Chennai, 600116, India, 91 9444368189; 2 Centre for Cyber Physical Systems, Vellore Institute of Technology, Chennai, India

**Keywords:** e-media, mobile software, home-based cardiac rehabilitation, cardiac rehabilitation, coronary artery bypass grafting

## Abstract

**Background:**

Noncommunicable diseases are a global concern with high mortality. Among these, cardiovascular disease requires more attention due to recurrence with altered physical activity. “SRCardioCare” (Sri Ramachandra Cardio Care) is an integrated mobile software that was developed to engage patients with effective communication and e-media support. We intend to explore the development of mobile software and its perceived impacts among health care professionals.

**Objective:**

This study aimed to develop a more economical and feasible platform for cardiac rehabilitation following conservatively managed coronary arterial disease, heart failure, postoperative adult cardiac surgical revascularization, and other cardiac surgeries, and to develop software that facilitates effective communication among participants and health care professionals.

**Methods:**

The software application was developed based on the experts’ interviews. The core components that are included in the software were assessed for their usefulness and applicability among people with cardiac disease using standardized questions. Physicians, nurse practitioners, and physiotherapists’ opinions were obtained. The developed app features providing e-media content for patients and pre- or postphysical activity response, including vitals and feedback from patients at set regular intervals, which were updated to the software.

**Results:**

Opinions obtained from practicing physicians (cardiologists), nurse practitioners, and physiotherapists were hopeful for the development and future implementation of “SRCardioCare” among patients. “SRCardioCare” is designed for the effective implementation of physical activity among patients after conservatively managed coronary arterial disease, heart failure, postoperative adult cardiac surgical revascularization, and other cardiac surgeries. An integrated communication medium and regular postphysical activity feedback of vitals may offer safety in implementing physical activity among the vulnerable in a remote setup.

**Conclusions:**

Remote rehabilitation is an essential and unexplored forum of practice in the field of rehabilitation, yet it requires wearable technology for remote monitoring and virtual reality and mixed reality for enhancing the adherence of the participants. To incorporate the telehealth forum effectively, especially in settings like India, the design must include an economically feasible and convenient model.

## Introduction

Technical support in the field of health care is an emerging trend fostering the development of telecommunication between specialists and other medical professionals with patients [1,2]. Owing to advancements in telemonitoring and telerehabilitation, various settings are being explored for diverse clinical conditions. Mortality rate due to noncommunicable diseases is represented at 72.3% globally, requiring closer attention toward the enhancement of prevention among the vulnerable population [3]. An important measure to be taken is to educate the patient on self-care and self-management strategies to prevent serious illness and its secondary complications. Satisfaction with doing physical activity among the patient population during the COVID-19 pandemic lockdown was high, and the telerehabilitation enhanced the patient-reported outcomes but was not limited to patient adherence [4,5].

Mobile software used in health care promotes inner strength and resilience among people with advanced age, which would be considered the core components of an individual’s health and quality of life [6]. Commercially available mobile-based software relies on behavioral and motivational theory, but very little literature exists for its applicability and success [7,8]. This encouraged health care professionals to implement physical activity with mobile health apps in enhancing physical health outcomes. Any intervention model shall satisfy the patient’s psychological well-being and meaningful choice, leading to sustained engagement in strategies implemented [9]. Data procurement using the software may require a hardware medium to source the most reliable and precise values during physical activities of an individual, which might act as a prime source for gaining confidence for one to actively use the software in remote settings [10,11].

Vital monitoring and supervised exercise program at remote settings would be a needed feature for prescribing the physical activity for an adult with cardiovascular disease, especially during postsurgical revascularization. Components with multiple domain-based software have appeared to be highly effective in engagement and the level of participation [12]. We aim to develop a mobile software that enables effective communication and feasible applicability, with safety measures incorporated for postsurgical revascularization patients, with provision for e-media–based exercise prescriptions. Patient adherence rate and rehospitalization, functional capacity, quality of life, and patient satisfaction would be analyzed after the effective implementation of the software application during hybrid cardiac rehabilitation.

## Methods

This was a qualitative feasibility trial, which was a part of a pilot trial before the main trial. An interface was developed for engaging cardiovascular disease patients and health care professionals with an effective mobile software–based technology, which was analyzed for its usefulness and applicability. Purposive sampling was performed among participants to reflect the exposure to cardiac rehabilitation enabled with technology, including physicians (n=5), nurses, cardiac intensive care nurses (n=10), and physiotherapists (n=15) of a total of 25 (Table 1). The systematic face-to-face interview was conducted, and the study was checked for its quality using Consolidated Criteria for reporting qualitative studies (COREQ) [13,14]. The responses received from the health care professionals are stated using the LIKERT scale. Software application would include medication adherence, wound care education, nutritional education, exercise counseling and training, 24/7 communication with the health care team, and associated telemonitoring with postexercise feedback.

The procedure of the experiment trial includes that the intervention group and control group would be trained for physical activity as per routine hospital protocol. Following traditional care, the interventional group would be introduced to a software interface for training, and the control group would receive training based on the routine phase II center-based rehabilitation (Table 1). The intervention group would be trained specifically for regular practice of “physical activity at home” with a safer and effective mode using prescribed dosage. Training would be provided to the participants for recording their vitals, including heart rate, oxygen saturation, blood pressure, and rating of perceived exertion, and they would be trained for documenting these in the software interface.

**Table 1.** Baseline characteristics of interview participants

Characteristic	Doctors	Nurses	Physiotherapists
Sex			
Male, n (%)	4 (80)	2 (20)	9 (60)
Female, n (%)	1 (20)	8 (80)	6 (40)
Age (years), mean (SD)	42 (5)	37 (12)	37 (8)
Experience, mean (SD)	16.8 (10)	14.9 (13)	15 (8)
Occupation	Cardiologist (n=2) Physicians (n=3)	Assistant nurse (n=6) Clinical in-charge (n=2) Senior nurse (n=2)	Clinical therapist (n=8) Senior therapist (n=7)

### Education

The management of the postoperative recovery period after a cardiovascular incident would be challenging, requiring an advanced approach for management due to various postoperative complications (Table 2). SRCardioCare enables patients to be effectively educated, where information about conditions, risk factors, and secondary prevention as well as exercise counseling and training is provided through video presentations. Telemonitoring data were collected from the patients, along with postexercise feedback and regular reminders of medications, including communication with the patient.

**Table 2.** An outline implementation of software to the participants with a follow-up for 12 weeks.

Module	Week 1‐2	Week 3‐4	Week 5‐6	Week 7‐8	Week 9‐10	Week 11‐12
Introduction and orientation	✓					
Training to self-evaluate vitals	✓					
Evaluation for risk stratification	✓					
Counseling - Medication		✓				
Counseling - Nutrition		✓				
Counseling - Functional capacity		✓				
Counseling - Setting a goal		✓				
Counseling - Physical activity			✓			
Re-evaluation - Functional capacity			✓			
Re-evaluation – Recovery vitals				✓		
Counseling - Energy conservation				✓		
Counseling - Relaxation and coping strategies				✓		
Counseling - Secondary prevention strategies					✓	
Counseling - Behavior changing techniques					✓	✓
Counseling - Leading a quality and comfortable life						✓

### Narrative View of Feasibility Checking Among Health Care Professionals

**Figure 1.**
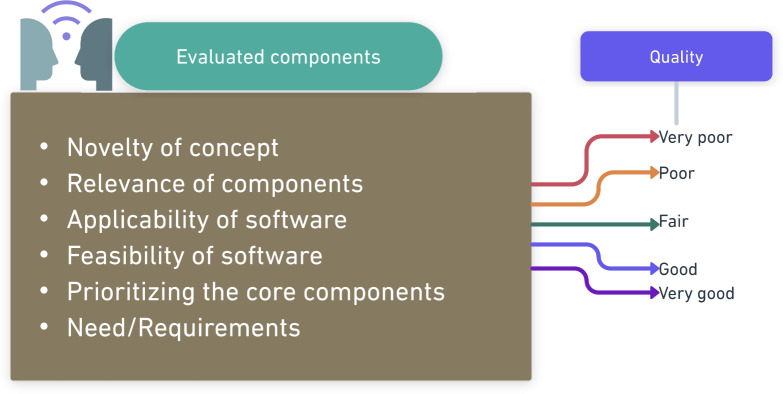
Survey among health care professionals for assessing the quality of software.

A systematic interview was conducted among various health care professionals using the following domain (Figure 1), which was stratified from the interview guide. Data were collected from the participants using descriptive field notes and interviews, which were repeated once after the required corrections were made based on feedback provided by the participants in the software. Literature supports that there have been deprived scenarios for implementing the physical activity following the cardiac events, including postoperative cardiac revascularization surgeries and other cardiac diseases. Since understanding the trends falling toward the implementation of newer interventions in health care, most of the health care workers agreed to develop the software. The following feedback was added to the prototype design as suggested by participants:

1. Components of psycho-social counseling
2. Nutritional education components
3. Behavioral change techniques
4. Ensuring safety for implementing physical activity in remote settings

### System Implementation

To build this mobile app for the rehabilitation of patients, Flutter was used to build the frontend and user interfaces, Node and Express for the backend, and MySQL as the primary database.

### Technology Stack

#### Front-End Development With Flutter

The frontend of the SRCardioCare app has been entirely made using Flutter—a UI framework developed by Google to create cross-platform apps. Some of the advantages of Flutter include the following:

1. Cross-platform support: Flutter can compile to both iOS and Android, our primary targets for this app, from a single code base, maintaining and updating new features with a small development team.
2. Developer experience: Flutter is paired with Dart, a language convenience that was high with building and debugging code, especially on physical devices. The hot reload allows us to observe changes live and assist in preventing the reinstallation of the app frequently.
3. Ecosystem: the ecosystem of packages and libraries available in Flutter. Some of those libraries include: ‘fl_chart’ for visualizations and ‘chewie’ for video playback.

### Interface Design

The user interface (UI) of SRCardioCare focuses on simplicity and accessibility, ensuring that patients with varying levels of tech literacy can navigate the platform with ease. The UI comprises several key features:

#### Dashboard

**Figure 2.**
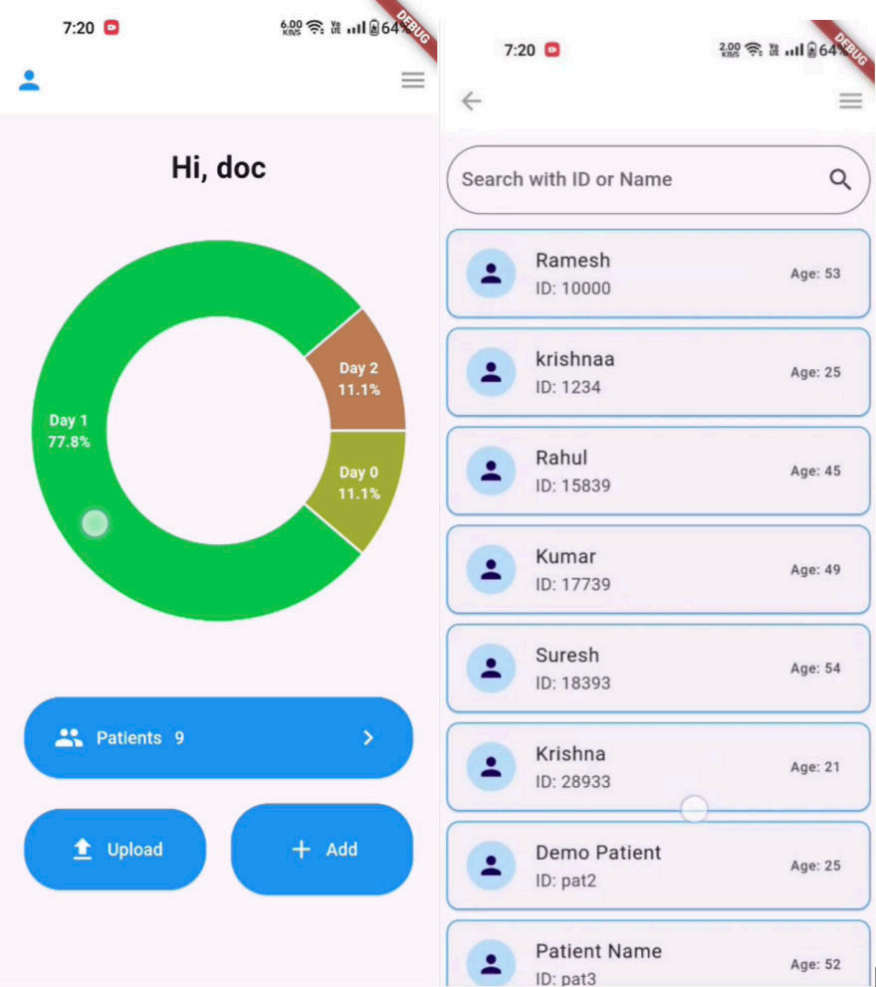
Developed software design home page for doctors with uploading videos options and patient progress.

A personalized dashboard is given for every user who logs into the app, for both patients and physicians (Figure 2).

- Patients: The dashboard shows the average of their vital parameters during their course of therapy. Links to all important actions such as viewing the current day’s exercises, help, and contact information of their dedicated physician are featured.
- Physicians: The dashboard shows a visualization representing the different days different patients are in; it provides them with a broad overview of their patients’ progress and offers them the ability to add a new patient.

#### Video Playback

**Figure 3.**
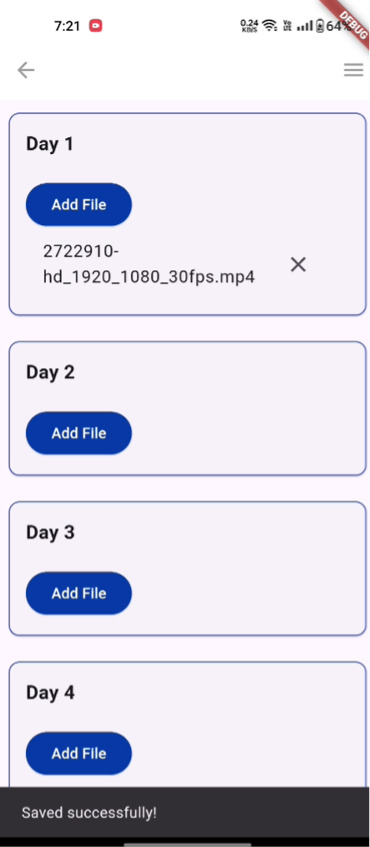
Uploaded videography of the exercises prescribed for the patient.

The Flutter `chewie` package on top of a video controller was used to present the video playback of the exercise videos uploaded by the Physician (Figure 3). Each patient can only view the video prescribed to them, in order, and only on the specific day selected by the Physician. This ensures patients cannot share sensitive information, and their physician can always update the exercise based on the feedback from the patient.

#### Feedback

**Figure 4.**
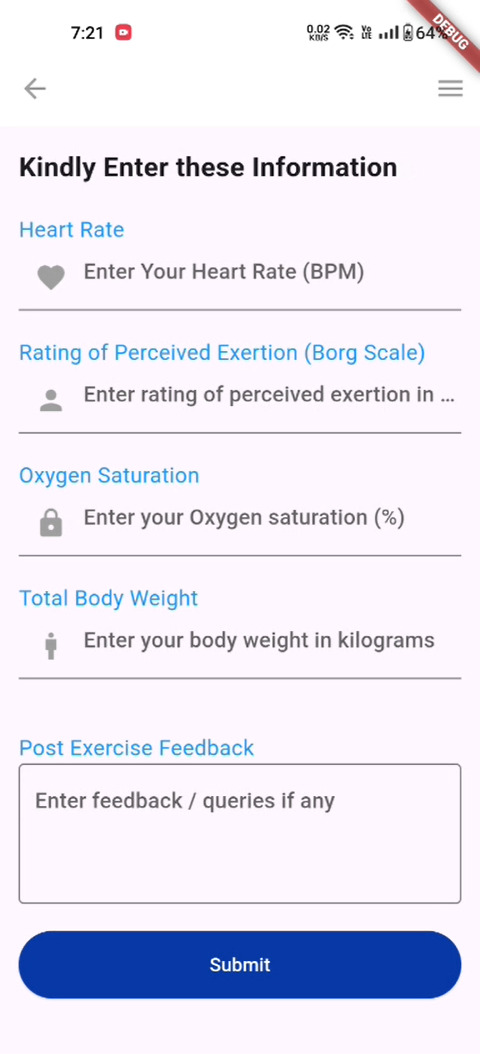
Documentation of post-physical activity vitals for patients.

After the patients complete the day’s exercises, they are provided with a form to input their vital parameters, such as heart rate, rating of perceived exertion, oxygen saturation, total body weight, and any remarks or doubts (Figure 4). This allows us to create a valuable record of their parameters throughout their journey and helps the physician tailor the exercise program further.

#### Technical Challenges

The primary challenge faced when developing this system was integrating both the frontend and backend together with APIs. We used a simple REST (Representational State Transfer) setup, but for safety, we implemented the interfaces of the models and data both in TypeScript and Flutter independently. The difficulty level is high due to Flutter’s rigid typing system and inability to convert JSON directly to appropriate types in Flutter. The manually written parsers for each model were used.

#### Backend

The backend was developed with Express; it runs using a bleeding-edge runtime called Bun. We preferred Bun instead of Node because of the following advantages: The typescript support directly runs a typescript file without having to transpile it to JavaScript. In HTTP/ Web APIs, the performance of Bun appears hastened compared to Node. Bun offers a great standard library, providing useful functions like hashing, which was used to verify our passwords on authentication.

Example- Bun hashing:

*let* isValid=await Bun.password.verify (password, user[0].password)

### Data Storage, Privacy, and Management

Currently, SRCardioCare uses MinIO, a S3-compatible block storage service that is self-hosted. This was used to store the videos uploaded by the physicians. It is a drop-in replacement for AWS S3; therefore, no code changes are required when moving the data. According to the Healthcare Data Protection Regulations by HIPAA (Health Insurance Portability and Accountability Act) and GDPR (General Data Protection Regulation), a clear consent would be obtained from the participants, data usage would be strictly limited and restricted, and participants would be given full rights to remove and access their data as required.

Advantages are that the files are isolated in different buckets, and the access control is strictly granted based on the rules from the database; access is restricted to patients and physicians for maintaining the integrity of data.

The access is limited based on the mechanism of presigning URLs, ensuring no patient ever has permanent access to the videos and preventing them from sharing the exercises without a physician’s prescription. Timely backups of the data for redundancy using external scripts such as *cron* and volume backups provided by the Cloud Provider could be scheduled.

### System Integration and Module Development

The emerging growth of coronary artery disease and Acute Coronary Syndrome needs an efficient rehabilitation technique that satisfies the desire of affected patients. Traditional methods have more limitations, including monitoring the patient’s risk when they are away and the need for continuous personalized care. This design addresses the above-mentioned limitation by supporting a personalized rehabilitation module and assisting them in a fast recovery from the disease. The proposed design methodology is a real-time cloud-based design to continuously monitor the patient and collect all the vital data of the patient.

The main goal of this proposed mobile app is to analyze and manage risk factors to reduce disability, improve functional capacity, lessen activity-related adverse effects, educate patients, and improve the quality of life.

To support the above-mentioned goal, the following design principles are adhered to: designed specifically for individual patients, collect vitals, processed offline, and prepared into a report specific to patients; development of a mobile application integrating technological advancements and cloud computing into health care, and continuous tracking of the patient based on the exercise.

### Ethical Considerations

Ethical clearance was obtained from the Sri Ramachandra Ethical Committee IEC/24/AUG/187/26. The registration number for this trial is CTRI/2025/03/082747. This study is a part of a randomized controlled trial, in which the developed SRCardioCare app would be implemented to compare the effects of home-based cardiac rehabilitation with center-based cardiac rehabilitation for adult surgical revascularization patients (CTRI/2025/03/082747). Written informed consent was obtained from all participants prior to enrollment. Participants were informed about the purpose of the study, the voluntary nature of participation, and their rights to decline to answer any questions or withdraw from the study at any time without any consequences. All interview data were deidentified prior to analysis. Personal identities were removed and confidentiality was maintained through secure, password-protected data storage accessible only to the research team. Participants did not receive any financial or material compensation for their participation in the study.

## Results

### Principal Results

**Figure 5.**
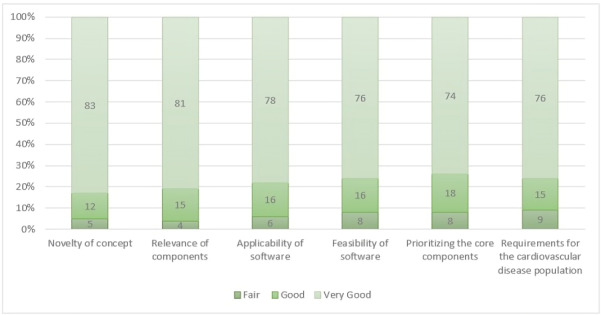
Responses from health care professionals: the built software was introduced to health care professionals in Sri Ramachandra Hospital and Medical Center, including 5 physicians, 10 nurse practitioners, and 15 physiotherapists. Responses were obtained using survey questions.

After the presentation of the developed mobile software (app) SRCardioCare, with various expert health care professionals, a total of 25 participants were interviewed for their opinion about the developed software. The acceptability of developed software from the professionals was high due to its application of multidisciplinary approaches, along with high relevance and clinical need (Figure 5). UI-enabled ease toward navigation to their dashboards and exercise videos without difficulties. Respondents provided positive feedback about the app and highlighted their satisfaction with features such as comments after exercises and vitals documentation. But vital documentation emerged as a poor efficiency due to varying levels of digital literacy among participants. Workflow issues addressed by the clinician would be time constraints in managing and replying to the vital details and comments provided by the participant's end.

- **Acceptability:** Health care professionals described the app-based platform as highly acceptable due to its integration with technology advancements and applicability in cardiac rehabilitation. The novelty of such innovation with tele-rehabilitation contributed to its acceptance.

*“The app is found useful for cardiac rehabilitation, especially for the heart failure population…”*

- **Usability:** Participants perceived the app as easy to navigate with its simplified dashboards and order of sequence for training without hurdles. Order of week-wise module with training videos supported hassle-free provision for participants.

“The *instruction provided in the app makes easy navigation in the app, providing good follow-ups…”*

- **Satisfaction:** Health care professionals expressed a completeness in app features that include a multidisciplinary counseling forum and improved patient engagement. The essential feature considered mandatory would be the acquisition of the post-vital monitoring and communication module by health care professionals.

*“I personally felt safe suggesting the patient get enrolled in cardiac rehabilitation using this software…”*

- **Barriers:** The manual entry of vital signs by the participants could affect the accuracy and compliance, as per the current trial protocol. Unless a monitoring device is prepared, the defects might be considered noncompliant. Technical challenges in linking the frontend and backend components were described.

*“Preloaded video components were slow occasionally and could reduce the medical terminologies used…,*

*The app could also be used for heart failure, where more precautionary measures need to be taken...”*

- **Facilitators:** The components, which included educational and personalized training, motivated health care professionals to prescribe the application. The visual dashboards and consistent communication between patients and providers engage patients.

*“It is good that the app displays the number of days the participants have to work on… and the exercises provided were kept changing every week, making participants more involved…”*

- **Workflow Issues:** Data flow and coordination between modules were concerns of workflow-related issues, and manual data entry by the participants would be considered a difficulty, indeed an issue. The current software is limited to such a feature; the second phase of the current trial would be an innovation of a real-time monitoring device that would gather data automatically from the participants' continuing exercise training. The integration of real-time monitoring devices was recommended to enhance workflow efficiency.

*“The alerting features and regular reminders would enhance the participation of patients effectively if done properly…”*

*“Rather than doing exercises independently, this app helps participants to perform without missing the sessions…”*

Experts supported the SRCardioCare mobile application, emphasizing the necessity of the innovation, stating that telehealth adoption is rapidly increasing. Content analysis of health care professionals’ suggestions and feedback revealed that they valued safety as a major requirement, stressing the necessity of continuous monitoring and postexercise feedback by the enrolled participants toward cardiac rehabilitation.

### App Outputs

#### Personalized Treatment

After the presentation of the developed mobile software (app), SRCardioCare, with various expert health care professionals, a total of 25 participants were interviewed for their opinion about the developed software (Figure 1). Based on the vital measurements, the physician will prescribe personalized exercises suited for each patient (Figure 3). The exercises are uploaded to their login and assigned to that patient based on the patient ID. The physician will upload a sequence of six nondownloadable videos that comprise the exercises to be done and will change after four days. The patient can log into the app but can only view the first exercise, which is assigned to their ID. These exercises may vary after four days. The same progress can also be viewed from the portal.

#### Baseline Assessment of Patient

**Figure 6.**
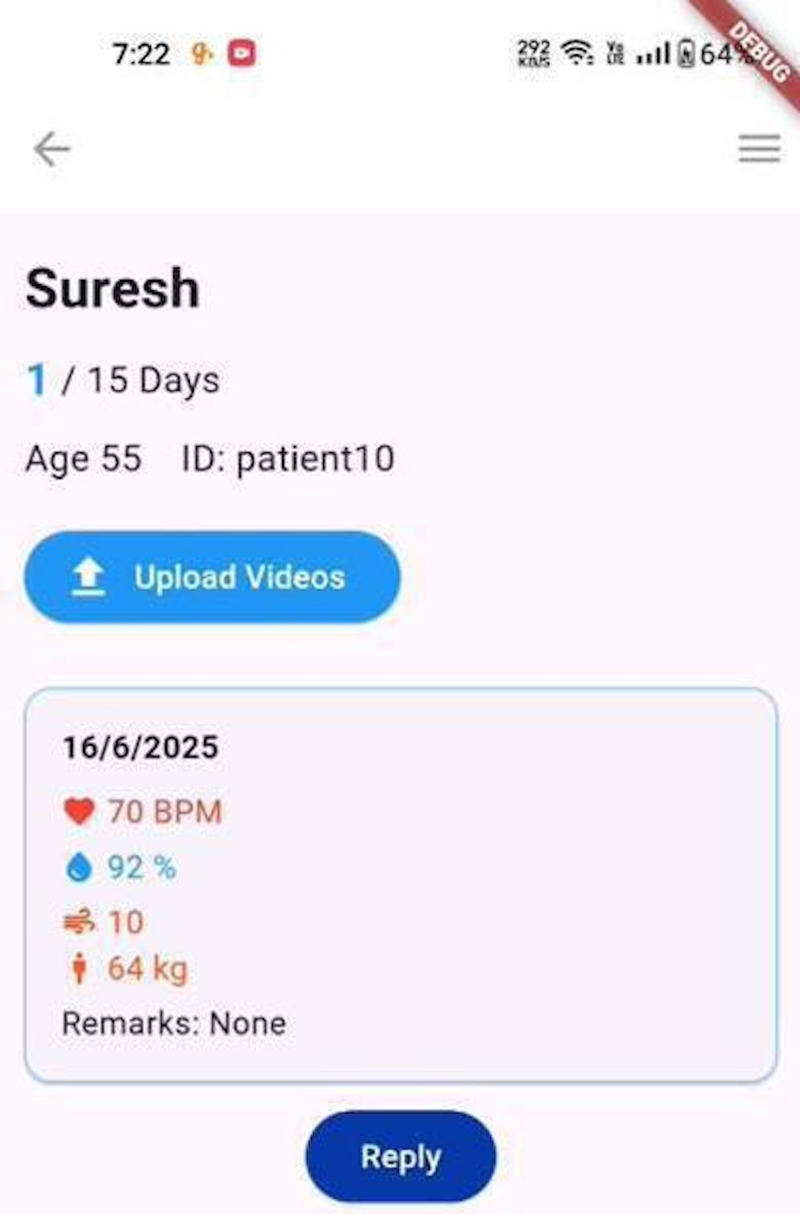
The screenshot shows the history of patients in the physician's login, along with the vital parameters measured.

This module is basic for the Remote Rehabilitation system. Before using this remote system (Mobile App), orientation for the patient is given on this app and the need of this mobile app and the postoperative self-evaluation, importance of their continuous monitoring of their vital parameters of the patient such as heart rate, oxygen saturation, rating of perceived exertion, total body weight, and provided ways of managing the risk in postoperative periods are explained (Figures 4 and 6).

**Figure 7.**
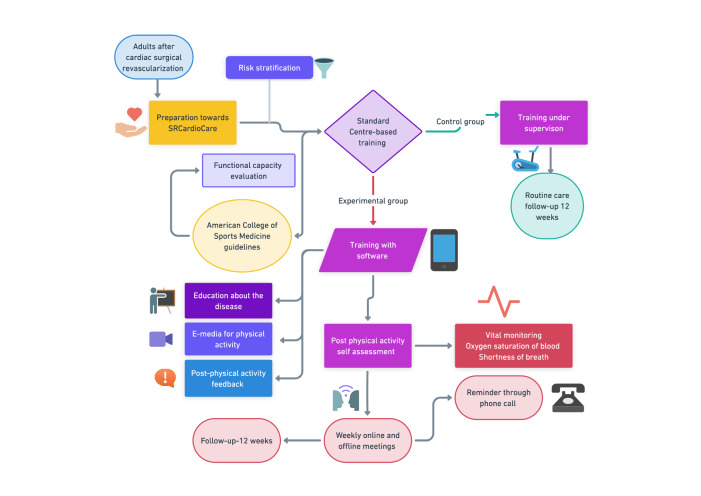
SRCardioCare Trial protocol for participants after introducing the software. The chart was developed using the Whimsical tool.

To support the patients for the above-mentioned program, a Mobile App was designed. The mobile app is facilitated with role-based access, such as physician and patient. A physician will be assigned to many patients. During the first two weeks of this program physician will record the vitals of the patient before they are discharged from the hospital, and based on the vital signs, a personalized rehabilitation program will be designed for the patient. When the patient returned home, they needed constant treatment with remote monitoring (Figure 7). The patient can log into the system and view the personalized exercises loaded to them by their physicians (Figure 3).

### Risk Factor Analysis

The main objective of this module is to assess the patient and provide continuous monitoring of their vital parameters. If needed, changes in the exercises can also be made. In the current app, the patient has to manually enter the vital parameter in the mobile app. As a future enhancement, it is planned to incorporate telemonitoring tools to record vital parameters. The vital parameters of the patients will be tracked and monitored by the physician. If any abnormality arises in the vital parameter, alert the caretaker or speed up the recovery process. This tracking prevents or reduces the adverse effects on cardiac activity.

This data is used to identify patterns and correlations between physical activity and vital signs, helping us assess the effectiveness of the rehabilitation plan and make informed adjustments. If a risk factor is detected (eg, prolonged elevated heart rate), the system sends alerts to both the patient and their health care provider, prompting immediate review and intervention. It also helps to predict the patient’s health before the risk arises.

### Data Analysis and Feedback Module

This mobile app establishes a communication between the physician and the patient at a remote setup. Through the app, the patient can readily present queries to the physician. They can record their vitals and post them to the physician. Physicians will resolve the queries raised by the patient on a priority basis. Physicians can analyze the patient’s vital data stored in the cloud and get detailed insights about the patient’s health, and they can predict the potential risk and avoid or reduce the adverse effects.

## Discussion

### Principal Findings

Cardiovascular disease remains the first among the major causes for years of life lost globally, ischemic heart disease being one of the causes if grouped globally [3,15]. The global risk of noncommunicable diseases is rising and requires immediate intervention to prevent complications [16]. Face-to-face implementation of exercises for chronic disabilities such as low back pain did not prove to be more effective than an exercise-based mobile app for managing the patients’ issues, according to a recent randomized controlled trial [17]. Decisions toward the development of a newer approach to deliver effective telemedicine and telerehabilitation are highly warranted [18]. A trial shows that intervention using a mobile app resulted in promising outcomes, stating that enhanced vitals, exercise capacity, and satisfaction toward exercise participation [19]. Device-dependent cardiac rehabilitation program for patients enhances the effective implementation of the protocol, as well as participants’ interest toward participation is also promisingly enabled [20].

Behavioral change techniques would be an emerging intervention that would initiate the hopeful scope toward physical fitness in society. The awareness should be obtained at the guardian level and the care provider level to ensure the implementation of physical activity among the vulnerable population [21]. A systematic review reveals that the mobile app–based intervention would enhance diet, physical activity, and sedentary behavior when applied modestly [12]. Another systematic review suggests that despite strong evidence, people fail to follow the suggested physical activity and guidance from clinicians to lead a healthy life [22].

Cardiac rehabilitation has been prescribed to patients after cardiac events, but effective implementations are becoming poor due to poor participation. Which might require an alternative method of implementation, including technology-enabled implementation of cardiac rehabilitation at remote setups as well as in-center setups [23-25]. Effective implementation of a telehealth program depends on the various domains that are to be incorporated to gain effective clinical outcomes for the participants under safer practice. That might include telemonitoring, medication adherence, education, self-awareness about their functional capacity, understanding, and mastery of the telehealth model for self-implementation by the patients [26,27].

Patients would be thoroughly evaluated by a health care professional before introducing the software app, and the assessed comorbid conditions would be considered for exercise prescriptions. Prior training would be provided based on the suggestions provided by physicians. The concepts providing the e-supported rehabilitation criteria would include implementing cardiac rehabilitation through an economically cheaper method. Henceforth, the app usage would be based on subscription, which depends on the requirements of the duration of rehabilitation sessions suggested by physicians.

### Conclusion

Globally, the literature supports that remote rehabilitation for cardiac diseases is effective. A mobile app offers trustworthy standards for the long-term adherence of patients with noncommunicable diseases. The developed software may help us to build a protocol for implementing remote rehabilitation following cardiac surgeries, heart failure, and conservatively managed acute coronary syndromes. Further upgradation may include telemonitoring support using the hardware gadget that would be designed to provide precise data of vitals during and post-exercise sessions. This leads the researchers to focus more on areas of telemonitoring devices that are more feasible and economical; deficient procurement of telemonitoring would be a limitation of this study.

## Abbreviations

ACS: acute coronary syndrome
CAD: coronary artery disease
NCD: noncommunicable diseases
UI: User Interface
